# Draft Genome Sequence of *Aquitalea magnusonii* Strain H3, a Plant Growth-Promoting Bacterium of Duckweed (*Lemna minor*)

**DOI:** 10.1128/genomeA.00812-17

**Published:** 2017-08-17

**Authors:** Hidehiro Ishizawa, Masashi Kuroda, Michihiko Ike

**Affiliations:** Division of Sustainable Energy and Environmental Engineering, Graduate School of Engineering, Osaka University, Osaka, Japan

## Abstract

*Aquitalea magnusonii* strain H3 is a promising plant growth-promoting bacterium for duckweed. Here, we report the draft genome sequence of strain H3 comprising 4,750,601 bp in 73 contigs. Several genes associated with plant root colonization were identified.

## GENOME ANNOUNCEMENT

Duckweeds (*Lemnaceae*) are tiny floating aquatic plants that can be utilized for phytoremediation as cleanup agents and for animal feed and biofuel production (1). Efficient cultivation of these aquatic plants can be achieved by the use of plant growth-promoting bacteria, and it has been shown as an effective and sustainable technology (2, 3).

In our previous study, *Aquitalea magnusonii* strain H3 was isolated as one of the dominant members of the bacterial community found on the surface of a duckweed (*Lemna minor*) (4). It was found that the strain H3 promotes the growth of *L. minor* by colonizing to the plants (4). Compared to the other plant growth-promoting strains isolated in the same study, it is noticeable that strain H3 exerted its ability of growth promotion irrespective of the presence of other bacteria (4). Although those features are probably due to the efficient colonization onto the duckweed roots and/or strong plant growth-promoting activity of strain H3, the molecular mechanisms of such aspects of this strain remain unknown.

The genomic DNA of strain H3 was extracted and purified using an illustra bacteria genomicPrep mini spin kit (GE Healthcare, Little Chalfont, United Kingdom) according to the manufacturer’s instructions. The genomic DNA was fragmented and sequenced by 101-bp paired-end sequencing with the HiSeq 2500 sequencing system (Illumina, San Diego, CA, USA), which generated 44,596,272 reads totaling 4,504 Mb. The sequences were assembled using *de novo* sequence assembler software (Velvet v. 1.2.08). The draft genome of strain H3 contains 73 contigs, which account for a total of 4,750,601 bp (59.4% G+C content), with an *N*_50_ of 177,045 bp and a maximum contig size of 501,139 bp. Gene prediction and functional annotation were performed with Rapid Annotations using Subsystems Technology (RAST) (http://rast.nmpdr.org/) and resulted in the prediction of 4,368 coding sequences (CDS), 5 rRNA genes, and 80 tRNA genes within the genome.

Several genes associated with the establishment of plant root colonization were identified in strain H3, such as genes for flagellar biosynthesis (*flg*), chemotaxis (*cheA*, *cheB*, *cheR*, *cheV*, *cheW*, *cheY*, and *cheZ*), aerotaxis (*aer*), type IV pilus biogenesis (*pil*), and biofilm adhesin biosynthesis (*pga*). The strain H3 is also equipped with genes involved in metabolisms of various organic acids, carbohydrates, and aromatic compounds, suggesting its potential to grow in the plant rhizosphere by utilizing these exudates from plant roots. Some plant growth-promoting traits are also implied by the presence of genes related to auxin biosynthesis and phosphorous supply (e.g., exopolyphosphatase and inorganic pyrophosphatase). The presence of these genes may offer valuable clues to increase understanding of the mechanisms of root colonization as well as the growth promotion by this strain.

### Accession number(s).

The draft genome sequences of *A. magnusonii* H3 have been deposited at DDBJ/EMBL/GenBank under the accession number BDST00000000.
